# Assessment of Occlusal Contacts Recorded with the Medit Intraoral Scanner vs. Exocad Software

**DOI:** 10.3390/jcm14207378

**Published:** 2025-10-18

**Authors:** Diana-Elena Vlăduțu, Răzvan Mercuț, Marius Ciprian Văruț, Alexandru Stefârță, Veronica Mercuț, Alexandra Maria Rădoi, Mihaela Roxana Brătoiu, Angelica Diana Popa, Adrian Marcel Popescu, Ștefana Dică, Răzvan Sabin Stan, Daniel Adrian Târtea

**Affiliations:** 1 Department of Prosthodontics, University of Medicine and Pharmacy of Craiova, 200349 Craiova, Romania; diana.vladutu@umfcv.ro (D.-E.V.); veronica.mercut@umfcv.ro (V.M.); alexandra.radoi@umfcv.ro (A.M.R.); smpopescu@mail.com (A.M.P.); 2 Department of Plastic Surgery, University of Medicine and Pharmacy of Craiova, 200349 Craiova, Romania; 3 Department of Biophysics, University of Medicine and Pharmacy of Craiova, 200349 Craiova, Romania; 4 Department of Dental Technology, University of Medicine and Pharmacy of Craiova, 200349 Craiova, Romania; alexandru.stefarta@umfcv.ro; 5 Doctoral School, University of Medicine and Pharmacy of Craiova, 200349 Craiova, Romania; dr.diana_popa84@yahoo.com (A.D.P.);; 6 Department of IT, University of Medicine and Pharmacy of Craiova, 200349 Craiova, Romania; 7 Department of Oral Rehabilitation, University of Medicine and Pharmacy of Craiova, 200349 Craiova, Romania; daniel.adrian.tartea@gmail.com

**Keywords:** occlusal analysis, Medit i700 intraoral scanner, digital impressions, DentalCAD Exocad software

## Abstract

**Background/Objectives**: Occlusal analysis is an important component of oral rehabilitation with a determining role in the prognosis of restorations. Over time, several qualitative and quantitative occlusal analysis methods have been proposed, starting with occlusion wax up to the most advanced digital systems. The objective of the present study was to evaluate and compare the data obtained through dental occlusion analysis using the Medit i700 and Exocad Elefsina v3.2 in a group of subjects, in order to establish the reliability or compatibility between the two occlusal analysis systems. **Methods**: The study was conducted on 20 subjects, aged between 24 and 53 years, who presented in the Dental Prosthetics Clinic of the University of Medicine and Pharmacy of Craiova. Digital impressions were acquired using the Medit Link v.3.3.6 intraoral scanner, and the digital files were subsequently uploaded from the Medit i700 into the Medit Occlusion Analyzer application and the Dental CAD Exocad software. For the analysis of occlusion in dynamics, mandibular movements and data acquisition, positions of edge-to-edge in protrusion, edge-to-edge in right laterotrusion and edge-to-edge in left laterotrusion were recorded, using the corresponding print screens. The 2D occlusal contact images generated by the two software programs were converted into .jpeg format and subsequently imported into Adobe Photoshop CS6 (2021) for comparative analysis. The data were statistically processed for each software used and the obtained data were subsequently compared. **Results**: The occlusal surfaces recorded with the Medit Occlusion Analyzer application represent 94% of the occlusal surfaces recorded with the Exocad software for the maxilla and 90% of the occlusal surfaces recorded for the mandible. In maximum intercuspation, the highest values were recorded by the Medit i700 software, whereas in edge-to-edge protrusion and both right and left edge-to-edge laterotrusion positions, the highest values were reported by the Exocad software. The discrepancy between maxillary and mandibular values arises from the conversion of the data from a three-dimensional to a two-dimensional format during image processing. **Conclusions**: The occlusal areas recorded by the DentalCAD Exocad software show higher values than those provided by the Medit Link software with the Medit Occlusion Analyzer application. The differences in recorded values, in the case of the digital flow of prosthetic restorations, require the intervention of the dentist to perform clinical adjustments to optimize occlusal relationships after the fabrication and cementation of restorations.

## 1. Introduction

In recent decades, dental practice has undergone a real revolution due to the introduction of digital tools that can be used to improve diagnosis and treatment methods [1]. Digital dentistry encompasses a wide range of technologies and techniques, among which three-dimensional (3D) imaging, computer-aided design and manufacturing (CAD/CAM), intraoral scanners and dental lasers are of great interest. These have improved the precision and accuracy of dental procedures [2]. The introduction of intraoral scanners (IOS) and advanced manufacturing processes, such as CAD/CAM technologies and 3D printing, has allowed the use of innovative dental materials for prosthetic restorations, providing the chance to replace conventional metal structures and improving the biomimetic and aesthetic results of restorations [3]. Optical impressions with IOS offer advantages such as reduced gag reflex, shorter working time, elimination of material-related distortions, and enhanced communication with patients and laboratories, thereby streamlining clinical practice [4]. Furthermore, the remarkable mechanical characteristics of these new-generation materials have allowed dentists to reduce the biological sacrifice of dental tissues, performing the procedure in a more conservative manner [5].

Intraoral scanners are used to obtain digital impressions of teeth and soft tissues, in order to create high-precision 3D virtual models of the oral cavity, which can be used for the design and production of dental restorations [2]. Thus, information is transmitted about the 3D shape of the prepared tooth, as well as adjacent and antagonist teeth, allowing for subsequent CAD/CAM processing of the prosthetic restoration [6]. In addition to digital images of the intraoral situation, intraoral scanners offer the possibility of recording occlusal relationships for quantitative analysis [7,8,9]. Such intraoral scanners include CEREC Ominicam, 3Shape-TRIOS and Medit. Medit Occlusion Analyzer is a more recent application developed by Medit Link to analyze dental occlusion, for diagnostic purposes and for reproducing the occlusal relationships of virtual maxillary and mandibular models. Occlusal analysis for prosthetic restorations is also performed by CAD applications using other specific occlusal analysis software, such as Exocad. CAD software creates occlusal morphologies based on conventional shapes that require substantial occlusal adjustments [10,11]. The ability to reproduce a patient’s dynamic occlusion in CAD is very important, but remains problematic because there are few methods available to record the complex and individualized dynamic occlusion and mandibular movement of a given patient. Therefore, CAD/CAM restoration design generally considers only static occlusion or the maximum intercuspation position (MICP) relationship, rather than dynamic occlusion [12].

Dynamic occlusion is generally reconstructed using a virtual articulator and CAD software, which simulates the movement of the mandible and visualizes the occlusal contacts [13,14]. Various methods can be used to simulate the movement of the mandible with a virtual articulator [12,15]. CAD software reconstructs the dynamic occlusion without taking into account the individual peculiarities of the temporomandibular joints (TMJ) and the mandibular mobilizing muscles.

The objective of this study was to evaluate and compare data obtained through dental occlusion analysis using the Medit i700 IOS and Exocad software for 20 subjects in order to establish the reliability or compatibility between the two occlusal analysis systems.

The null hypothesis assumed that there is no statistically significant difference between the results provided by the two software in terms of occlusal analysis.

## 2. Materials and Methods

### 2.1. Study Design

The present study was conducted within the Dental Prosthetics Clinic of the University of Medicine and Pharmacy of Craiova, during the year 2025. Twenty subjects, patients of the Faculty of Dentistry Clinic aged between 24 and 53 years, of both genders, were selected, who had their digital impressions taken with the Medit i700 intraoral scanner, for occlusal analysis. After taking the digital impressions, the digital files were uploaded from the Medit Link software to the Medit Occlusion Analyzer application and the Dental CAD Exocad software.

The minimum number of participants was computed using the software application G*Power 3.1.9.7, Heinrich Heine University Düsseldorf, Germany, based on matched pair comparisons, the power defined as 1 − β equal to 0.8, a significance level α of 0.05, and a medium effect size value (taking into account the fact that there are not many data available in the literature, and focusing on practical significance), thus resulting in a study lot of 19 participants.

The study was approved by the University and Scientific Ethics and Deontology Committee of the University of Medicine and Pharmacy of Craiova No. 234 of 7 December 2022 and was conducted in compliance with the Declaration of Helsinki of 1975.

The inclusion criteria were subjects of both genders with intact dental arches, with permanent dentition, without the presence of the third molar and without signs of temporomandibular dysfunctions.

The exclusion criteria were subjects with edentulous gaps, subjects with prosthetic restorations or orthodontic appliances, and subjects with cooperation difficulties.

All subjects were trained on the intraoral scanning procedure for obtaining virtual models. The purpose and objectives of the study were also explained and informed consent was obtained. Before the intraoral scanning, an exo-oral and endo-oral clinical examination was performed on each subject, followed by professional intraoral hygiene.

After scanning the dental arches, the dental occlusion was recorded in the MICP, as well as the protrusion, right and left laterotrusion movement, according to the protocol for using the Medit i700 scanner. All subjects presented normal occlusal contacts, without orthodontic anomalies.

For intraoral digital impression recording, the Medit i700 intraoral scanner with the Medit Link v.3.3.6 application (Medit, Seoul, Republic of South Korea) was used, and for occlusion analysis, the Medit Occlusion Analyzer application and the Exocad Elefsina v3.2 design software (Darmstadt, Germany) were used.

Medit i700 technical specifications: accuracy for the entire arch: 10.9 μm ± 0.98, scanning frame up to 70 FPS; imaging technology 3D in motion video technology/3D full color streaming capture; adaptive anti-fogging; it is able to automatically assess the occlusal contacts between the two dental arches and illustrate the results through a color map.

### 2.2. Medit i700 Scanning Technique

Calibration was performed before the start of the scanning session according to the manufacturer’s instructions. For intraoral scanning, subjects were seated in the dental chair with their heads in extension of the spine.

A latex-free lip and cheek retractor (Optragate 3D) was used to facilitate easy access to the oral cavity, with the soft tissues being evenly spaced around the mouth, for isolation and moisture control.

The scanning began with the maxillary arch. The tip, a component of the handpiece that includes the intraoral camera, was inserted into the oral cavity, and the recording began with the occlusal surface of the last molar of the right hemiarch. It continued with slow and constant zigzag movements, following the shape of the arch. After the last molar on the left hemiarch was scanned, the scanner was oriented at 45 degrees towards the vestibule and the scanning continued from posterior to anterior carefully, in order to scan 2–3 mm of the gingival mucosa [16,17]. This was followed by scanning the vestibular and oral surfaces of the arch. A similar procedure was followed for scanning the mandibular arch.

To position the two virtual maxillary and mandibular models in occlusion, the intraoral camera was inserted on the vestibular surface, distally, the patient being asked to remain in the MICP. The scan was performed by zigzag movements in the bilateral lateral area.

For dynamic occlusion scanning, mandibular movements and data acquisition, recordings of the edge-to-edge position in protrusion (EEPP), edge-to-edge right laterotrusion position (EERLP) and edge-to-edge left laterotrusion position (EELLP) were made, for which the corresponding print screens were created.

### 2.3. Processing Digital Files in Exocad

Exocad is currently one of the most well-known prosthetic restoration design programs used in dentistry. It was created in a limited version for dental offices under the name Exocad ChairsideCAD and in a complete version for Dental Technology Laboratories under the name Exocad DentalCAD. In the present study, Exocad DentalCAD, version 3.2 Elefsina, was used.

In the basic version, Crown&Bridges, DentalCAD offers technicians the opportunity to design a series of aesthetic and functional dental restorations. Depending on the design needs of laboratories, Exocad offers the possibility of expanding the basic program with a series of modules with which complex dental restorations can be made. In addition to the basic function of designing dental restorations, Exocad comes with a series of functions that have the role of analyzing imported virtual models before starting the actual design.

Visualizing intersections/distance to antagonists or adjacents.

**Figure 1:**
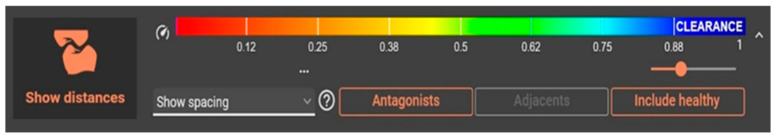
The “Show distances” function in Exocad.

The function that allows analyzing the contacts, intersections and proximities of restorations to antagonists and adjacent teeth is “Show distances” (Figure 1).

This function allows the analysis of virtual models resulting from the intraoral scan for the study, offering the possibility to visualize the intersection (interpenetration) between the two arches (Show interference contacts); to observe the intersection as well as the spacing between the slopes of the two arches (Show spacing); and, depending on the set distance, to display the contacts similar to occlusion paper (Show contact areas).

For this study, the Exocad base module known as “Crown&Bridges” was used, accessing only the function for analyzing virtual models that were imported in their original unmodified form from the Medit Link software.

**Figure 2:**
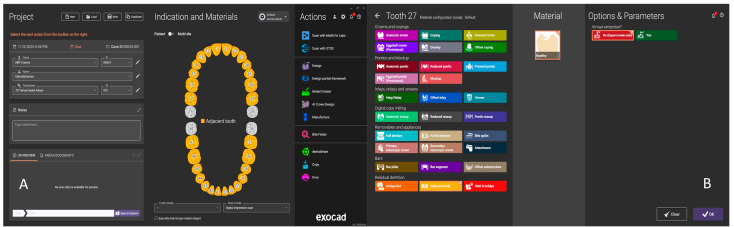
(**A**) Patient’s file in Dental DB. (**B**) Defining the status of teeth.

In the first stage, the patient’s file was completed in Dental DB (patient database, Figure 2A), where both arches with existing healthy teeth were selected as “Healthy” (Figure 2B), so that the software would not propose design steps in Dental CAD, but would only allow the import of the two antagonist arches in occlusion.

**Figure 3:**
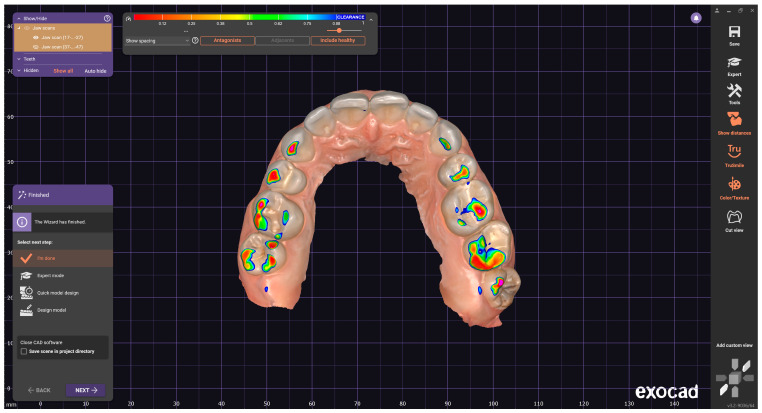
Dental CAD, displaying contacts in MICP on the maxillary arch.

The next step consisted of importing the two arches in the MICP, then activating the “Show distances” function, activating “Hide” for the mandibular arch, so that the contacts could be observed on the maxillary arch (Figure 3). This procedure was repeated by importing the mandibular arch in the EEPP, EERLP and EELLP, so that the contacts were displayed on the maxillary arch depending on the type of movement. To visualize the mandibular contact surfaces, the same procedure is followed, keeping the mandibular arch visible and hiding the maxillary arch (“Hide”).

In the present study, the contacts between the two arches were defined by a preset color scale from the “Show spacing” function. In the Medit Occlusion Analyzer application of the Medit Link software, the color scale featured blue, cyan, green, yellow, and red, where blue signifies a reduced contact and red a close contact (collision, interpenetration). For the Exocad design software, the color scale varied between magenta and blue, in a color range between 0 and 1, where magenta (0) signifies collision, and the spacing between the slopes of the two arches appears from 0.5 (green) on the color scale to blue (1) (Figure 1). The acceptable tolerance zones were highlighted in green. It can be seen that, although the two software are complementary and compatible, they can serve for occlusal analysis, but the data provided differ.

**Figure 4:**
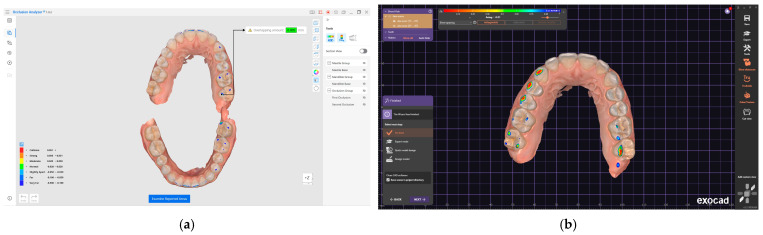
(**a**) Medit Link color histogram. (**b**) Exocad DentalCAD color histogram.

Therefore, for the present study, the surfaces from blue to red are considered for the Medit software, and for the Exocad software, the colors from magenta to green are used (Figure 4a,b).

### 2.4. Image Processing Chain

The 2D occlusal contact area images obtained using the two software were converted to .jpeg format and then transmitted to Adobe Photoshop CS6 2021 (Adobe Systems, San Jose, CA, USA) for comparison, a software used for editing digital images. For each subject, the pixel number in the contact areas of the two arches was obtained through image processing and subsequent analysis in the graphics processing program. The technique has also been used in other studies [16].

The images obtained from the two software were edited by discarding areas of no interest and resized, and the resolution was adjusted to equalize the variations resulting from image acquisition with the two software (Medit Occlusion Analyzer, Exocad DentalCAD). The selection of the color range was made with a tolerance of 1–5%.

To extract the weight of a particular color, color selection software was used and the colors from the analyzed range were selected one by one. For Medit Occlusion Analyzer, the colors blue, cyan, green, yellow and red were analyzed, with the meaning of contact (from reduced to collision), and for Exocad DentalCAD, the colors magenta, red and yellow were analyzed, with the same meaning, specifying that after the acceptable tolerance zone represented by the color green, on the color scale, the meaning is the spacing between the slopes of the two arches.

**Figure 5:**
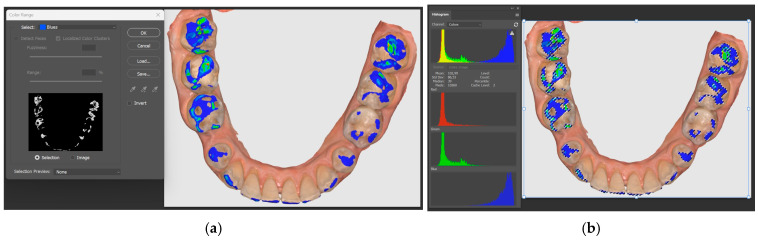
(**a**) Selecting the color of interest. (**b**) Pixel identification.

After selection, it was possible to evaluate the histogram separately for each image (Figure 5a,b), collecting in the central table the number of pixels corresponding to the total surface of the colors in the contact areas, for the position of MICP, EEPP, EERLP and EELLP for each subject.

### 2.5. Statistical Analysis

The primary data resulting from the occlusal recordings in this study were centralized in a table using Microsoft Excel 365 (San Francisco, CA, USA). The data were statistically processed for each software used and the obtained data were subsequently compared using IBM’s Statistical Package for the Social Sciences (SPSS), version 26.0 (IBM Corp., New York, NY, USA). Continuous variables were expressed as mean ± standard deviation (SD), for the ease of presentation, as well as medians, and their normality was verified using the Shapiro–Wilk test. The Sign test was used to compare the matched observation for all participants. The value 0.05 was chosen as statistical significance threshold. The Microsoft Excel application was used to obtain data presentation graphs, included in the Section 3.

## 3. Results

Given that the data provided on the color scale by the two software depending on the degree of occlusal interpenetration are different, the total contact surface between the two arches was analyzed.

**Figure 6:**
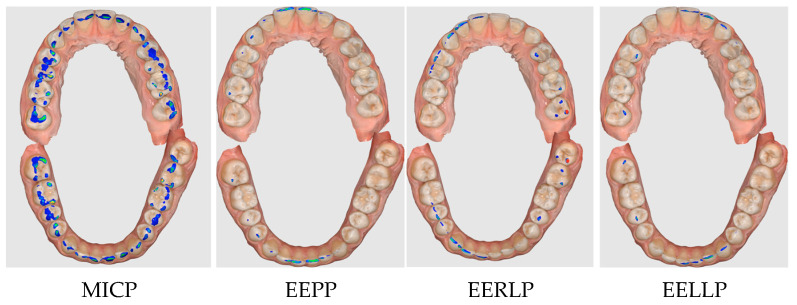
Aspects of Medit i700 recordings for MICP, EEPP, EERLP, EELP (the color scale features blue, cyan, green, yellow, and red, where blue signifies a reduced contact and red a close contact (collision, interpenetration)).

**Figure 7:**
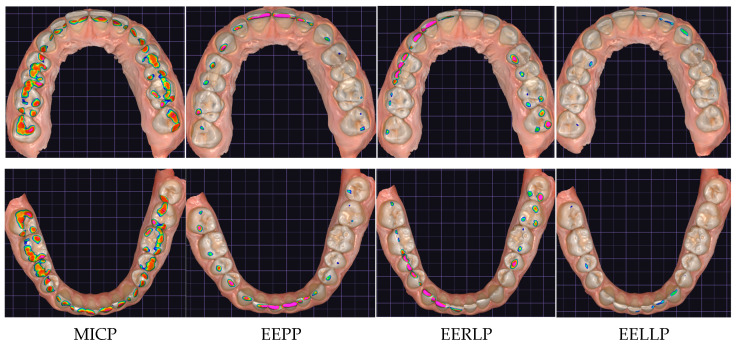
Aspects of records in DentalCAD Exocad (allows only alternative presentation of arches). The color scale varies between magenta and blue, in a color range between 0 and 1, where magenta (0) signifies collision, and the spacing between the slopes of the two arches appears from 0.5 (green) on the color scale to blue (1).

The results obtained by the two occlusal analysis methods reveal data regarding the dimensions of the contact surfaces between the two arches in the positions of MIC, EEP, EERL and EELL. Aspects of the recordings of these positions are shown in Figure 6 (Medit i700) and Figure 7 (DentalCAD Exocad).

### Analysis of the Results Obtained Based on Occlusal Analysis with Medit Occlusion Analyzer and DentalCAD Exocad

Following the quantitative evaluation of the occlusal surfaces recorded by the two software, in the positions of MIC, EEP, EERL, and EELL, for the maxilla and mandible, a specific number of pixels was defined for all positions, for each participant. Comparing the data provided by the two software applications, based on the number of pixels, it is found that the sum of all occlusal surfaces recorded with the Exocad software (170,860 maxilla pixels, 187,640 mandible pixels) is higher than that recorded with the Medit Occlusion Analyzer software (160,957 maxilla pixels, 168,954 mandible pixels). The occlusal surfaces recorded with the Medit Occlusion Analyzer application represent 94% of the occlusal surfaces recorded with the Exocad software for the maxilla and 90% of the occlusal surfaces recorded for the mandible. The difference in values between the maxilla and the mandible results from the fact that through image processing, they were transformed from 3D to 2D.

**Figure 8:**
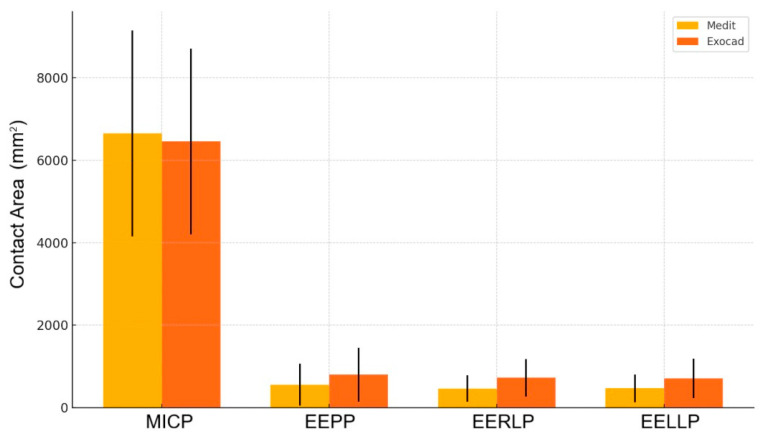
Comparative analysis of contact area measurements (MEDIT vs. EXOCAD). Maxillary average contact area plotted with error bars corresponding to SD.

Analyzing the recorded surfaces for each position of the mandible relative to the maxilla, it was found that in the case of the Exocad software, the surface recorded in MICP is smaller in the maxilla than that recorded with the Medit Occlusion Analyzer software; in the other positions, the surfaces recorded with Exocad have higher values than those recorded with Medit (Figure 8 and Table 1).

Based on the results of the Sign test for MICP, the test statistic, 0.258, is quite small, meaning that the difference between MEDIT and EXOCAD is minimal. The *p*-value, 0.798, is much higher than the common threshold of 0.05, indicating that the difference is not statistically significant. There is no strong evidence to suggest that MEDIT and EXOCAD have significantly different means. The observed difference is likely due to random chance, due to normal measurement variability, rather than a meaningful difference in measurement accuracy.

Regarding EEP position, both software applications yield similar values. Although EXOCAD has slightly higher mean values, the variation between subjects is too high to consider this a real difference. Clinically, both are comparable for this measurement. The differences observed between the two software applications are likely due to normal measurement variability, not to systematic bias.

Regarding EERL position, EXOCAD tends to systematically report higher values for lateral right contact area compared to MEDIT. This could indicate a difference in how the two applications segment or calculate contact area. Possible overestimation by EXOCAD (or underestimation by MEDIT) is indicated by these results. The inadvertence between EXOCAD and MEDIT could constitute a major problem in creating dental restorations that fit well with opposant teeth.

**Figure 9:**
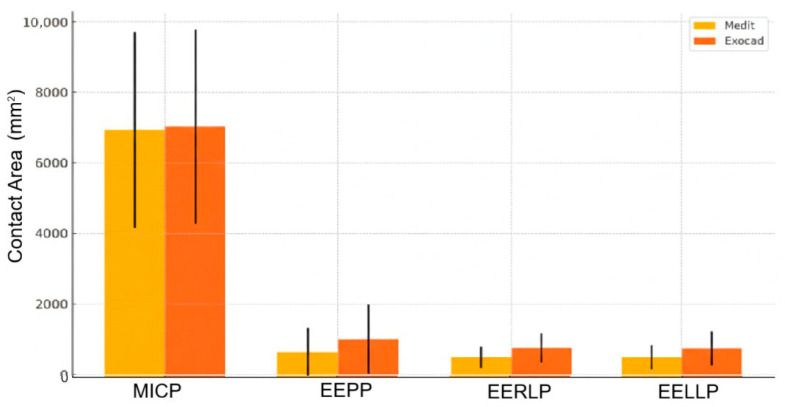
Comparative analysis of contact area measurements (MEDIT vs. EXOCAD). Mandible average contact area plotted with error bars corresponding to SD.

In summary, EXOCAD tends to report higher values for EEPP, EERLP, and EELLP. The error bars represent the standard deviations, quite large in some cases, which explains why many differences are not statistically significant. The biggest visible difference (and statistically significant) is for EERLP, consistent with the Sign test result (Figure 9 and Table 2).

**Figure 10:**
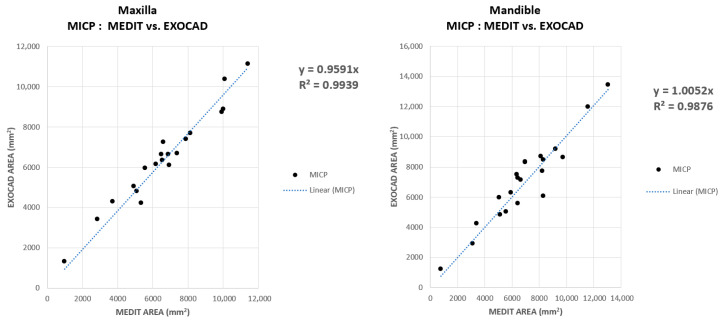
Comparative analysis of maxillary and mandibular contact areas for MICP in EXOCAD vs. MEDIT.

For each subject participating in this study, comparing the contact areas recorded for MICP in Exocad to those recorded in Medit, the graphic representation in Figure 10 resulted. In other words, within this graphic, the value for the contact area for MICP in the Exocad software is compared to that in Medit.

Each point in Figure 10 represents a subject for which a contact area was determined in MICP in Exocad and Medit respectively. On the vertical axis, generically called ordinate, are the values for Exocad, and on the horizontal axis, called abscissa, are the values for Medit. Based on the points created in this graphic representation, a linear regression with the null free term of the form y = a × x (null free term) was constructed. This linear regression has the role of numerically indicating the correlation of the values for a contact area determined in Exocad and Medit respectively.

The slope of the linear regression, denoted by the letter “a”, can be read on the linear regression equation on the side of the graph. A slope value equal to unity indicates similar values in Exocad and Medit. A slope value above unity indicates an overestimated value for the contact area in Exocad, and a sub-unity value indicates an overestimated area in Medit.

Another important parameter is the coefficient of determination, R2. This parameter of linear regression indicates the relevance and/or correspondence of the obtained results, in particular, the slope of the fitting line (of the linear regression). In other words, the closer the points in the graphical representation are distributed around the line obtained by linear regression, the higher the R2 is, approaching unity.

As can be seen in Figure 10, on the ordinate are the values for the contact areas in the MICP determined in Exocad. On the abscissa, expressed in mm2, as well as those on the ordinate, are the contact areas determined in Medit. The slope value for the linear regression is sub-unit in the graphical representation of the contact area for the MICP at the maxilla, this value indicating an overestimation of the contact area of the MICP in Medit. For the mandible, the slope value is very close to unity, which denotes that the areas obtained in Exocad are comparably equal to those in Medit. The high value of the coefficient of determination, R2, indicates a high correspondence of the slope for both the data associated with the contact areas in the MICP for the maxilla and for the mandible. Moreover, a uniform and compact distribution of points around the line associated with the linear regression is observed.

**Figure 11:**
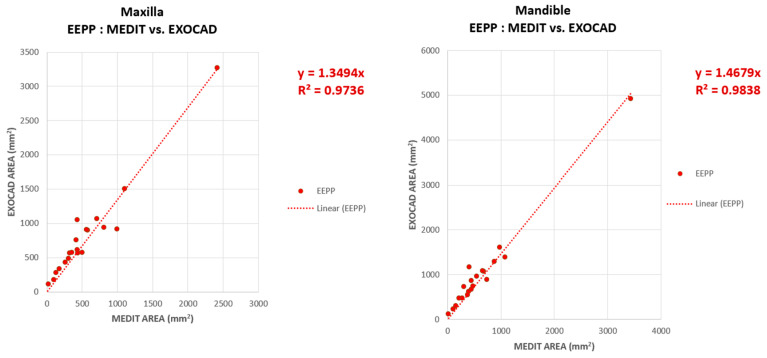
Comparative analysis of maxillary and mandibular contact areas for EEPP in EXOCAD vs. MEDIT.

The contact areas for the EEP position, determined in the two software subjected to comparative examination, Exocad and Medit, do not have a uniform distribution around the linear regression line, one subject having a high contact area for the EEPP. It can be seen in Figure 11 that the point with high coordinate values is located in the upper part of the graphical representation, both in the representation for the maxilla and for the mandible.

A high, super-unitary value of the slope can be observed in both the maxilla (1.34) and mandible (1.46) graphical representations, these results indicating an overestimation of the contact surface values for EEPP in Exocad. In other words, the values on the ordinate (contact area in Exocad) are higher than the values on the abscissa (contact area in Medit).

**Figure 12:**
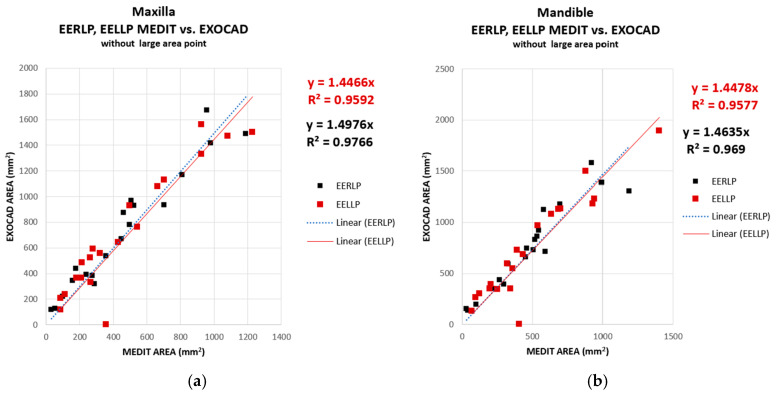
Comparison of the contact area measurements obtained using EXOCAD and MEDIT for the EERLP and EELLP. Plotted data—maxilla (**a**); mandible (**b**).

The graphical representations for the contact areas in EERLP and EELLP were merged to see if the two software considered in this study render the values for the areas in EERLP (black) and EELLP (red) similarly. As can be seen in Figure 12, the contact areas, for both EERLP and EELLP, present super-unitary values of the slope (in the range 1.44–1.49), which denotes that both areas in the lateral contact zones are overestimated in Exocad. The coefficient of determination exhibits a slightly lower value; however, it remains sufficiently high to indicate an adequate correlation of the results.

**Figure 13:**
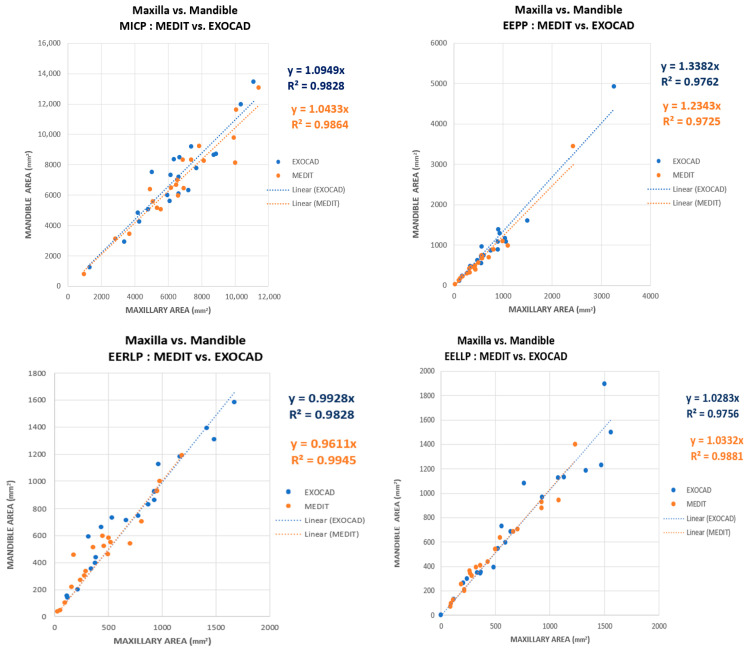
Corresponding contact areas from maxilla and mandible [EXOCAD (blue) and MEDIT (orange)].

Another data analysis performed in this study was to compare the contact areas obtained for the mandible (ordinate) with the contact areas obtained for the maxilla (abscissa). The results obtained for both software are shown in the graphic representation. In Figure 13 it is observed that the slopes of the linear regressions are close to unity for the MICP, EERLP and EELLP, but for the position of EEP the contact areas are overestimated for those associated with the mandible. This overestimation is valid in both Exocad and Medit.

The overestimation may be due to the points associated with high contact areas in the EEPP. All the plots in Figure 13 show a high degree of relevance. This is indicated by the high values of the coefficient of determination, R2.

To conclude, EXOCAD reports consistently higher values for EEPP, EERLP, and EELLP. The difference on EERLP remains visually and statistically the most pronounced. Error bars (standard deviations) are still quite large, which explains why many *p*-values are not significant.

## 4. Discussion

The study of dental occlusion represents an important stage in the dental disciplines and at the same time the study of dental occlusion has become a field of dentistry [18,19,20,21]. In a narrow form of the definition, the term “occlusion” refers to the arrangement of the maxillary and mandibular teeth and the way in which the teeth of the two arches come into contact [22]. In a broader context, however, the definition of the term “occlusion” is not limited to the morphological contact relationships between the teeth. Rather, it embraces the dynamic morphological and functional relationships between all components of the masticatory system—not just the teeth and their supporting tissues, but also the neuromuscular system, the TMJ, and the craniofacial skeleton [18,22,23,24].

Dental occlusion is one of the most debated topics in the literature. The Glossary of Prosthodontic Terms (GPT-10) defines occlusion as the static relationship between the incisal or masticatory surfaces of the maxillary and mandibular teeth [25]. However, this definition captures only one aspect of occlusion. In practice, occlusion encompasses dynamic activities such as mastication, swallowing, and bruxism, all of which subject dental restorations to continuous forces throughout their lifespan. According to Goldstein, the longevity of dental restorations depends on several factors, such as the materials used, patient-related factors, and dentist-related factors [26]. This broader perspective has had a significant impact on how dental practitioners approach occlusion considerations in treatment planning and restorations [27,28,29]. Clinicians who have this knowledge can better appreciate how restorations will perform under functional stress and can include occlusal adjustments as part of routine examinations and procedures [27].

Occlusion is closely related to oral health and is considered an important indicator of the functional status of the masticatory system [30,31,32]. Occlusal analysis plays an important role in the diagnosis, treatment and prognosis of prosthetic restorations [33,34], orthodontics [35,36,37], dental implants [38] and maxillofacial surgery [39]. When occlusal discrepancies are not addressed, they often lead to restorative failures and manifest as fractures, wear, periodontal complications or worsening temporomandibular dysfunction [40,41]. A comprehensive understanding of occlusal relationships is fundamental for minimizing the risk of long-term complications.

Each improvement in occlusal analysis methods has brought major changes in dental practice [16]. They should supply information regarding the location of occlusal contacts, their duration, and the magnitude of occlusal forces [30]. Dental occlusion is qualitatively assessed using occlusal materials (articulation paper, wax, or silicone) to document the positions of teeth and their antagonists. The limitations of these qualitative methods include the low reproducibility of the recordings and their inherent lack of objectivity. Quantitative recordings use various indices to assess dental occlusion. Among the digital occlusal analysis systems, studies have been published using the T-Scan III analysis system, the Dental Prescale system, the Blue Silicone system, the Medit i600 and CAD software [30,42,43,44,45]. In general, the content of occlusal analysis should include occlusal contact area (OCA), occlusal contact number (OCN), and occlusal force [30].

The objective of this study was to compare the correspondence of two digital methods of occlusal analysis, the Medit Link software with the Medit Occlusion Analyzer application and the DentalCAD Exocad software. Utilized for digital oral impressions, the Medit i700 intraoral scanner facilitates the alignment of two virtual models in occlusion, effectively capturing occlusal relationships. It further allows the analysis of occlusion by providing data on contact locations, surface areas, and interpenetration levels using a color-coded scale. The Exocad software also provides the same data, but uses a different color scale. To simplify the study and reduce errors for the recordings obtained with Medit, the entire surface (from blue to red) was taken into account, while for Exocad, the surfaces ranging from magenta to green were considered, where on the color scale, the transition beyond green corresponds to the appearance of the interocclusal space between the two arches. For both software, the highest occlusal contact values are recorded for MICP, but with large differences in terms of the occlusal contact surfaces.

The present study compared the occlusal surfaces corresponding to the MICP, EEPP, EERLP and EELLP recorded with the Medit i700 software and analyzed with the Medit Occlusion Analyzer application and DentalCAD Exocad software. The study revealed differences between occlusal contacts recorded by the two software, both between the maxilla and mandible, reflecting clinical practice where prosthetic restorations often require adjustments despite seemingly accurate adaptation in Exocad. To address this, Exocad enables digital adjustment of occlusal contacts between arches, reducing the need for clinical corrections.

An explanation could be given by the fact that the 3D images were transformed into 2D images by means of the Adobe Photoshop software and by the fact that there are differences between the data provided by the two software. Thus, with the exception of the position of the MICP in the maxilla, where the Medit Occlusion Analyzer application gave higher values than the DentalCAD Exocad software, for all other positions in the maxilla and mandible, the Exocad software revealed higher values. In current practice, all these inconsistencies are reflected in the prosthetic restorations through occlusal discrepancies. Several studies highlight the occurrence of discrepancies and emphasize the necessity of clinical adjustments by the dentist to achieve proper occlusal adaptation of the prosthetic restorations.

A study conducted for occlusal analysis and restorations [42] showed that 17.3% of restorations obtained based on CAD software (Dental system) were oversized, requiring subtractive occlusal adjustments, and 7.7% required additive occlusal adjustments; therefore, they were undersized. Risciotti (2024) even drew attention to the need for meticulous verification of clinical occlusion records after the realization of prosthetic restorations [42]. In another study, Yifan Zhang (2019), referring to FPDs (fixed partial dentures) made by digital flow, states that adjustments are necessary at the occlusal and interproximal levels [46].

A further study compared implant crowns fabricated using two workflows: fully digital (intraoral scanning + CAD/CAM) versus conventional (analog printing and traditional laboratory). The results showed that clinical adjustments were required for restorations fabricated using both workflows and that these were more significant in the conventional workflow. Adjustments were required for the interproximal and occlusal surfaces [47].

A review by Bohner in 2019 on the accuracy of intraoral scanners reported similar results [48]. Thus, scanning a dental arch using intraoral and laboratory scanners ranged from 17 μm to 378 μm. For edentulous arches, the scanners showed an accuracy ranging from 44.1 to 591 μm and between 19.32 and 112 μm for digital scanning of dental implants [48]. Based on our experience, we believe that inaccuracies occur both during intraoral scanning and during the technological flow. Several studies have evaluated the effects of occlusion on the durability of restorations. Prosthetic restorations that preserve the pre-existing occlusion exhibit superior longevity relative to those with overestimated (elevated) occlusion or occlusal interferences during excursive movements (horizontal), conditions that expose the restoration to unfavorable biomechanical forces [49].

The longevity of dental restorations is influenced by multiple factors, including the size of the restoration, the tooth’s position within the arch, the patient’s caries index, material selection, moisture control, and various other patient- and clinician-related variables [50]. Among these, occlusion remains one of the most important, yet often overlooked, factors that determine restoration failure. A lack of understanding of fundamental occlusal principles may result in ineffective trial-and-error approaches to managing patient complaints, ultimately compromising the outcomes of restorations [51]. This underscores the importance of appropriate occlusal analysis, both in the planning and execution phases of restorative procedures.

Therefore, it is essential to ensure that restorations integrate into the patient’s original occlusal pattern to minimize stress and prevent premature failure. Conversely, restorations with improper occlusal contacts can lead to localized stress concentration, resulting in microcracks or decimation over time [27].

In accordance with the results of this study, the null hypothesis was confirmed for three out of the four studied parameters, as there were no significant differences in the results provided by the DentalCAD Exocad software and those provided by the Medit Link.

Limitations of the study: In the comparative analysis of contact area measurements, Exocad software consistently estimated a larger contact area than Medit software across all examined positions. This discrepancy suggests that Exocad may employ a different computational approach or parameterization, leading to broader surface estimations. The observed differences highlight potential variations in algorithmic interpretations between the two software, which may impact clinical or technical assessments. Further investigation is necessary to determine whether these differences are systematic or influenced by external factors such as scanning resolution, software processing, or underlying geometric assumptions. Another limitation of the study is the potential discrepancies that appear by converting 3D data into 2D images using Photoshop.

## 5. Conclusions

The Medit Occlusion Analyzer application and the DentalCAD Exocad software are occlusal analysis methods that provide data regarding the location and size of occlusal contact surfaces.

The contact surfaces between the two arches presented the highest values in the MICP for both evaluation methods. The data provided by the DentalCAD Exocad software presents higher values than those provided by the Medit Link software with the Medit Occlusion Analyzer application.

This study underscores the occurrence of discrepancies in occlusion within the digital workflow of prosthetic restorations, drawing the attention of dental practitioners to potential clinical implications. In light of the variations in recorded values, it is imperative for the dentist to perform clinical adjustments to optimize occlusal relationships after the fabrication and cementation of restorations.

## Figures and Tables

**Table 1:** Evaluation of contact surfaces and differences between MEDIT and EXOCAD (in module), at maxillary level.

Parameter	Category	Minimum Difference	Maximum Difference	Mean ± SD	Test Statistic	*p* *
MICP	MEDIT	33	1204	6652.80 ± 2500.43	0.258	0.798
	EXOCAD			6453.45 ± 2257.96		
EEPP	MEDIT	64	842	557.65 ± 509.42	−1.291	0.205
	EXOCAD			797.55 ± 656.44		
EERLP	MEDIT	28	713	462.16 ± 325.01	−2.089	0.044 ^#^
	EXOCAD			724.11 ± 456.90		
EELLP	MEDIT	16	632	469.80 ± 335.80	−1.834	0.075
	EXOCAD			708.40 ± 475.07		

* Sign test. ^#^ Statistically significant.

**Table 2:** Evaluation of contact surfaces and differences between MEDIT and EXOCAD (in module), at mandible level.

Parameter	Category	Minimum Difference	Maximum Difference	Mean ± SD	Test Statistics	*p* *
MICP	MEDIT	17	2233	6927.95 ± 2781.62	−0.116	0.908
	EXOCAD			7029.55 ± 2757.06		
EEPP	MEDIT	92	1480	636.15 ± 699.30	−1.365	0.181
	EXOCAD			1002.45 ± 975.95		
EERLP	MEDIT	92	653	490.21 ± 303.84	−22.861	0.028 ^#^
	EXOCAD			751.79 ± 412.36		
EELLP	MEDIT	2	616	497.45 ± 335.84	−1.829	0.076
	EXOCAD			740.75 ± 489.64		

* Sign test. ^#^ Statistically significant.

## Data Availability

The authors declare that the data of this research are available from the corresponding authors upon reasonable request.

## Abbreviations

The following abbreviations are used in this manuscript:

CAD/CAM	Computer-aided design/Computer-aided manufacturing
IOS	Intraoral scanners
MICP	Maximum intercuspation position
EEPP	Edge-to-edge position in protrusion
EERLP	Edge-to-edge right laterotrusion position
EELLP	Edge-to-edge left laterotrusion position
TMJ	Temporomandibular joint
GPT-10	The Glossary of Prosthodontic Terms
OCA	Occlusal contact area
OCN	Occlusal contact number
